# Indications, Technique, and Outcomes of Patient Specific Instrumentation for Osteotomy About the Knee

**DOI:** 10.1007/s12178-025-09987-2

**Published:** 2025-07-09

**Authors:** Harmon S. Khela, Monty S. Khela, Varun Sriram, Grant G. Schroeder, Ian Hollyer, Seth L. Sherman

**Affiliations:** 1 Perelman School of Medicine, University of Pennsylvania, Philadelphia, PA 19104 USA; 2 Creighton University School of Medicine, Omaha, NE USA; 3 Renaissance School of Medicine at Stony Brook University, Stony Brook, NY USA; 4 Department of Orthopaedic Surgery, Stanford University Medical Center, Stanford, CA USA

**Keywords:** Patient-specific instrumentation, Knee osteotomy, High tibial osteotomy, Distal femoral osteotomy, Mechanical alignment

## Abstract

**Purpose of Review:**

Osteotomies around the knee are well-established techniques used to correct lower-extremity malalignment. While osteotomies of the proximal tibia and distal femur have traditionally been performed free-hand, the advent of Patient-Specific Instrumentation (PSI) in the form of custom 3D printed cutting guides and implants offers surgeons a greater ability to individualize surgical corrections to a patient’s unique bony anatomy. This review aims to investigate the current state of the literature surrounding the use and outcomes of PSI for knee osteotomies and the benefits and drawbacks of PSI compared to traditional techniques.

**Recent Findings:**

Recent studies have demonstrated the potential benefits of PSI in knee osteotomy, including improvements in the accuracy of planned corrections, reductions in operative time and fluoroscopy exposure, and similar patient-reported outcomes. While increased costs and lead time represent drawbacks to the use of PSI, the technology continues to evolve such that these areas may improve over time.

**Summary:**

For osteotomy of the distal femur and proximal tibia, PSI offers surgeons an opportunity to improve surgical precision intra-operatively, with similar outcomes and complication rates as compared to traditional osteotomy techniques.

## Introduction

Abnormalities in lower limb alignment can lead to a variety of adverse outcomes, such as knee pain, instability, and an elevated risk for ligamentous injuries and osteoarthritis [1–4]. In order to correct lower extremity malalignment, osteotomies such as high tibial osteotomy (HTO) and distal femoral osteotomy (DFO) are well-established procedures that can be used to address single or multiplanar deformities around the knee [1, 4]. Knee osteotomy commonly corrects coronal plane malalignment to unload the medial or lateral compartments [5].

Although knee osteotomies are generally associated with good results, they can be technically challenging and have known complications, including fractures, neurovascular injuries, delayed or non-unions, or undesired final alignment correction [6–9]. When deformity analysis is based on pre-operative assessment with 2D radiographs, variations in patient positioning may also lead to inaccurate intra-operative assessments and differences between the planned and post-operative corrections [10–12]. Accurate assessment of deformity in these operations is critical, as over or under-correction can result in inferior outcomes, including recurrent deformity or accelerated degeneration in another knee compartment [10, 13–15].

In order to increase the precision and efficiency of knee osteotomy, patient-specific instrumentation (PSI) has emerged as a promising tool [16, 17]. PSI typically uses pre-operative volumetric imaging with low dose computed tomography (CT) or magnetic resonance imaging (MRI) to generate 3D models of the patient’s anatomy while also often incorporating X-rays in the templating. While other templating technology, such as intra-operative computer-guided navigation, relies on expensive equipment with is susceptible to malfunctioning electronics and often longer operative times, PSI represents a relatively simple modification of existing techniques [18]. PSI and custom guides and models give surgeons intra-operative templates to specify angular corrections with high precision and potentially offer advantages over traditional freehand techniques. This review aims to highlight the current state of literature surrounding the use and outcomes of PSI for knee osteotomy and limb deformity correction.

## Pre-Operative Planning and Creation of PSI

**Fig. 1:**
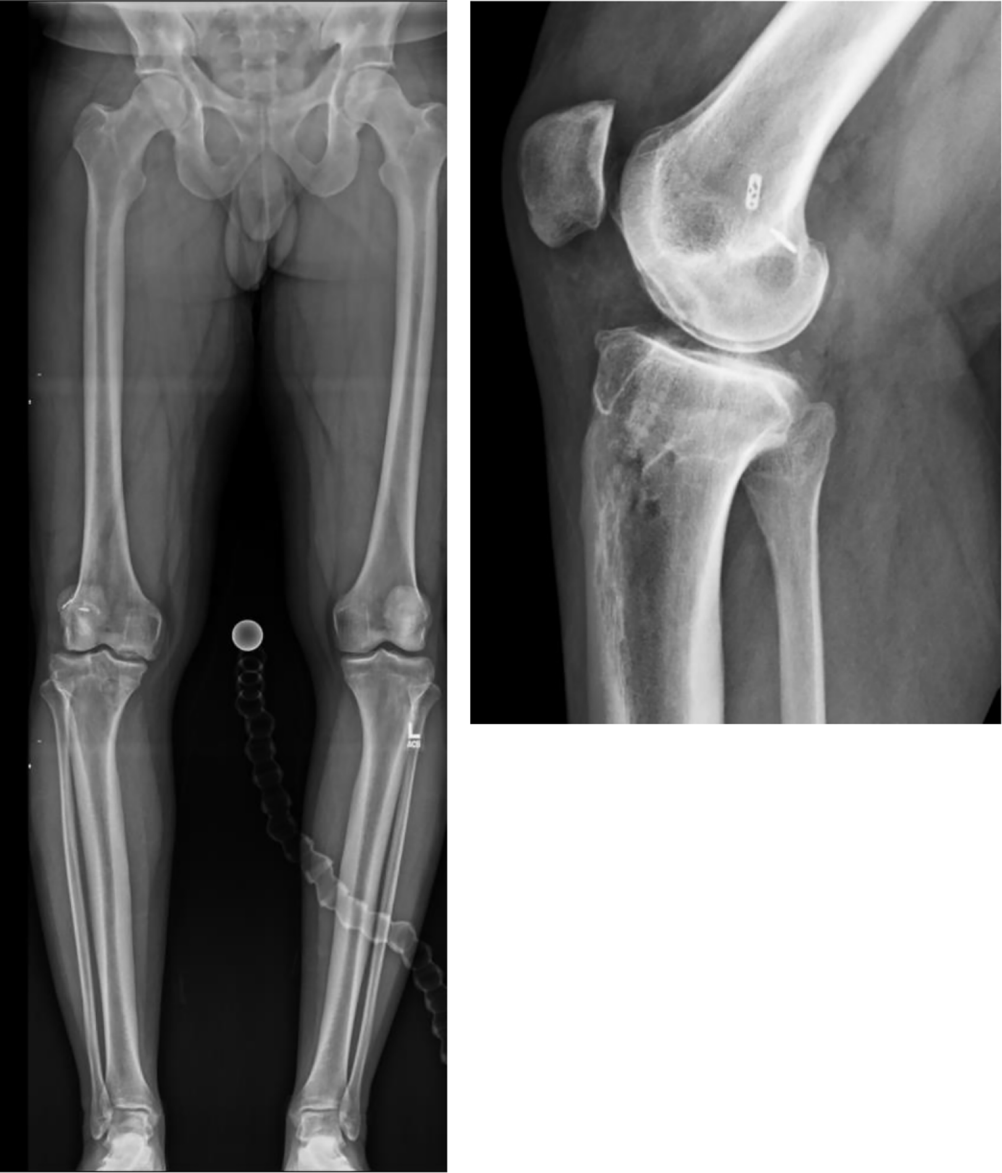
Full-length anteroposterior mechanical axis view and a knee lateral radiograph of a 25-year-old patient with right knee in varus alignment with excessive tibial slope, with a history of ACL reconstruction with allograft and meniscal repair, complicated by graft rupture, ACL tunnel bone grafting and osteochondral allograft to his medial femoral condyle, revision ACL reconstruction with hamstring autograft, and a subsequent re-rupture

When assessing a patient with lower extremity deformity, initial evaluation involves obtaining a weight-bearing full-length mechanical axis view radiograph of the lower extremities to visualize the hip, knee, and ankle in the coronal plane for deformity analysis (Fig. 1), in addition to other views as needed (weight bearing anteroposterior, posteroanterior flexion, lateral, and Merchant views of the knee, or lateral tibial standing X-ray if evaluating for tibial slope).

Historically, osteotomies for the lower extremity were planned using only 2D radiographs [19]. However, variables such as flexion, limb rotation, and weight bearing have been reported to cause inaccuracies when using 2D imaging to plan deformity correction, especially when more than one plane may be involved [12, 20–23].

Volumetric imaging of the lower extremity, knee joint, or at least the proximal tibia if a multiplanar osteotomy is planned, is then obtained. The foundation of PSI in knee osteotomies is 3D volumetric imaging, such as CT or MRI. Some surgeons prefer CT scans over MRI because they are less expensive, have shorter acquisition times, can often be scheduled more quickly, and provide better resolution of bony anatomy [24]. While the downside of CT is ionizing radiation, modern, effective radiation doses can be reduced to the equivalent of one full-leg standing radiograph using low-dose scanning protocols and comes with the potential benefit of less fluoroscopy needed intra-operatively [24].

**Fig. 2:**
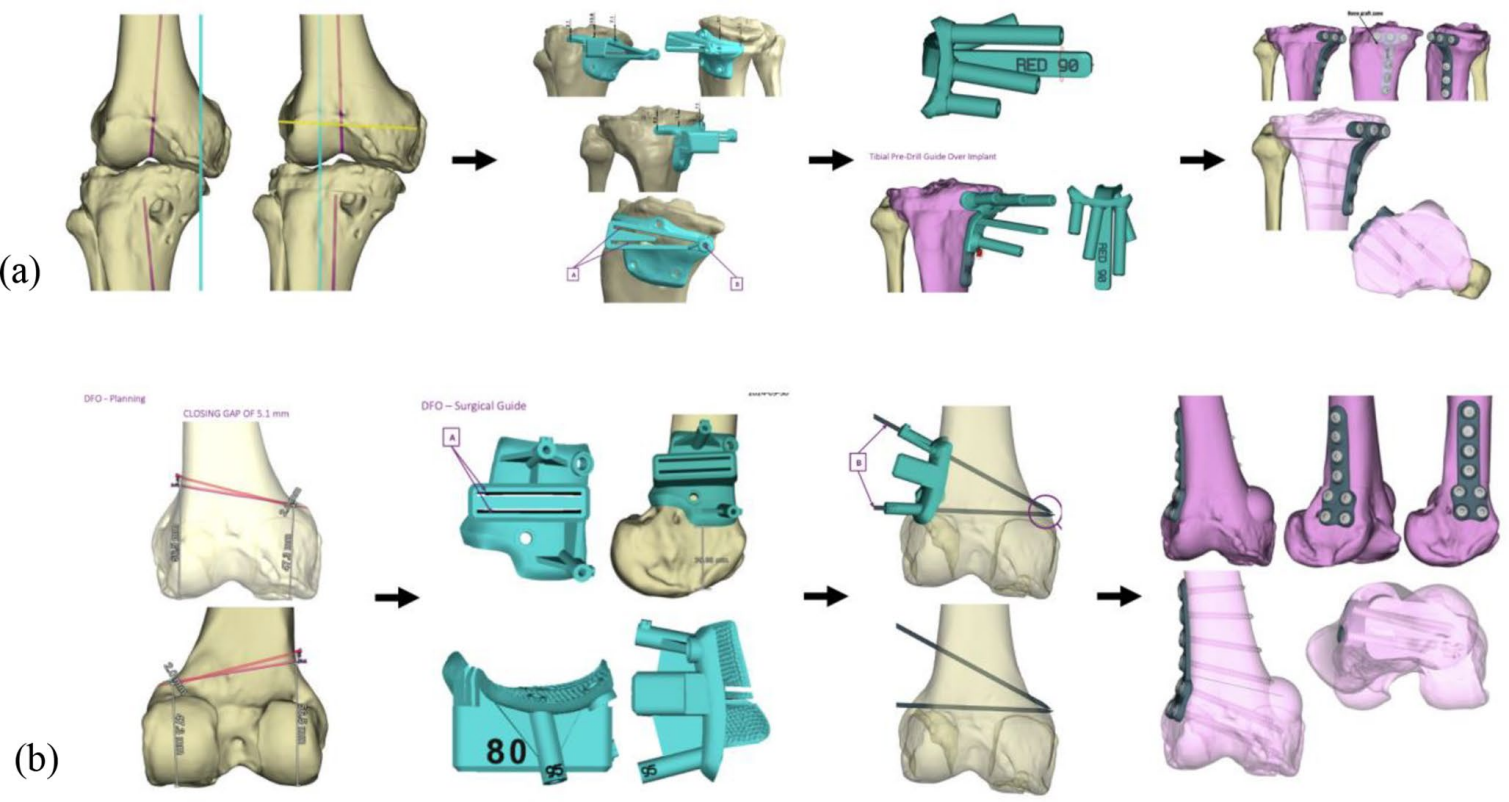
The patient from Fig. 1 was templated to correct (**a**) varus with a femoral closing angle of 5.0° and gap of 5.1 mm with a lateral closing wedge distal femoral osteotomy with lateral fixation, and (**b**) a tibial slope correction of 10.7° and 10.7 mm with an anterior closing wedge high tibial osteotomy with medial fixation

**Fig. 3:**
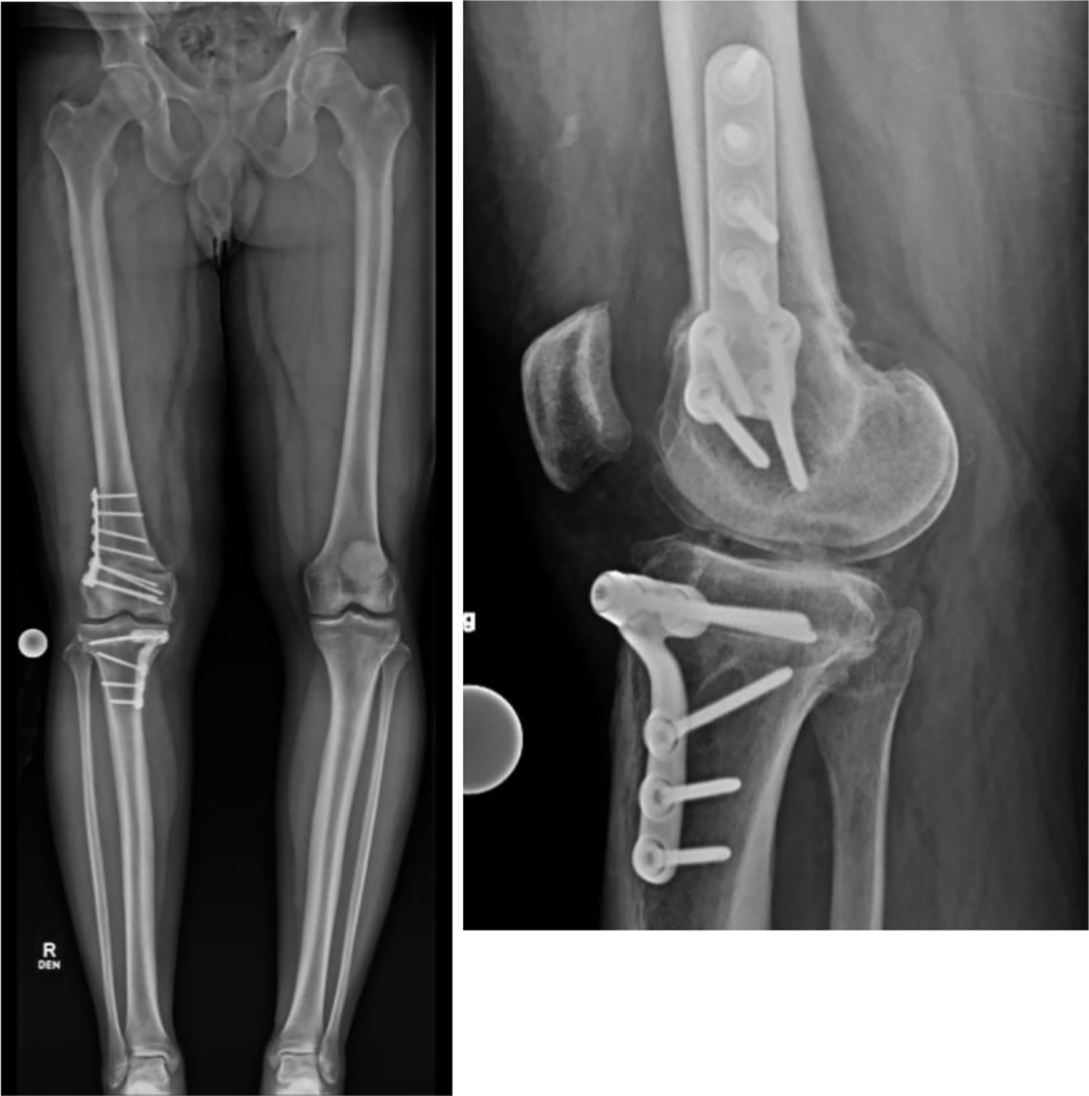
Final full-length anteroposterior and lateral radiographs of the patient from Figs. 1 and 3 months post-operatively

The 3D imaging files are then loaded into a software package for segmentation to create bone models and analyzed by 3-D planning software to create virtual pre-operative plans determining correction sizes and planned bone cuts approved by the surgeon [25, 26] (Fig. 2). The pre-operative templating software typically determines the exact length of each guide pin or screw, as well as plate size and shape, and can even take into account bone loss from the kerf of the saw blade [24]. 3D bone models, custom guides, and/or custom plates are made available to the surgeon intra-operatively to enable safe, efficient, and accurate plan execution (Fig. 3).

Deformity analysis during pre-operative planning for knee osteotomies is a multi-faceted process that requires consideration of various anatomical and biomechanical factors to optimize outcomes. Traditionally, the mechanical medial proximal tibial angle (mMPTA) has been a primary tool for pre-operative planning, as it focuses correction around the tibia without being influenced by patient positioning artifacts that may affect measurements like the mechanical femorotibial angle (mFTA) or weight-bearing axis [25, 27]. The mMPTA has also been shown in research to accurately correct limb alignment compared to other measures, such as the weight-bearing line ratio or joint line convergence angle (JLCA) [27]. Along with mMPTA, osteotomy planning should also involve consideration of the mechanical axis deviation (MAD), mechanical lateral distal femoral angle (mLDFA), JLCA, joint obliquity, and sagittal plane measures such as tibial slope and femoral version/tibial torsion. MAD represents the deviation of the mechanical load-bearing axis from the neutral limb alignment and is critical in determining the site of correction. When MAD falls within the metaphyseal regions of the tibia or femur, a single-level osteotomy can be sufficient. However, when MAD is excessively shifted outside the bone boundaries, a double-level osteotomy is often required to distribute the correction while maintaining joint congruency [28]. The location of correction is also influenced by whether the primary issue is pain or instability, with medial compartment osteoarthritis and isolated varus deformity often benefiting from HTO, while valgus malalignment and lateral compartment overload commonly involving DFO. Ligamentous laxity and other instability patterns further determine whether the correction should be performed at the femur, tibia, or both [29, 30], or involve soft tissue repair or reconstruction.

The decision to perform a single or double-level osteotomy is guided by the magnitude and location of the deformity. If a single mMPTA and mLDFA correction must go beyond 95° of mMPTA, this can result in excessive joint line obliquity and increase shear forces on the cartilage, potentially accelerating degenerative changes [31]. Additionally, the JLCA, which reflects coronal plane laxity, can influence whether bony correction alone is sufficient or if additional soft tissue balancing procedures are needed [32].

The choice between opening-wedge and closing-wedge osteotomies depends on the desired correction geometry, the patient’s bone quality, and soft tissue constraints. Open-wedge techniques may require grafting, while closing-wedge osteotomies may have a lower risk of delayed union. PSI has been shown to enhance both techniques by pre-determining wedge dimensions and optimizing hinge placement, which gives surgeons the ability to change both coronal and sagittal alignment with one cut which is easier to perform.

After virtual corrections have been planned, patient-specific cutting guides are designed and created. These are all custom tools tailored to fit the patient’s anatomy intra-operatively. Using the guides intra-operatively is as important as the virtual pre-operative planning, as research has shown that when virtual pre-operative plans are only used as a reference without printed guides, over 20% of patients have post-operative alignment > 3° outside the planned correction [33].

Optimizing the efficiency of image acquisition, processing, and PSI manufacturing is dependent on each surgeon’s practice environment. While some institutions can facilitate this process in-house with 3D core facilities, other surgeons may choose to use commercial companies to help facilitate the process, though this may increase overall costs [24].

Patient models are typically printed using medical-grade resin, polycarbonate, acrylonitrile butadiene styrene, polyethylene terephthalate glycol, and nylon-based materials [34]. The raw materials for PSI can be quite inexpensive, as some authors have reported printing PSI for less than €2.5-5 euro per patient, while an appropriate 3D printer may only cost €500–2000 euros. However, more expensive manufacturers can deliver higher quality guides and models [35, 36]. Ultimately, all PSI must be capable of withstanding the sterilization processes, such as steam pressure autoclaves, gamma rays, or low-temperature hydrogen peroxide sterilization [24].

### PSI Surgical Technique

Intra-operatively, patients are typically positioned supine on a radiolucent table to facilitate fluoroscopic imaging, with a lateral post used to stabilize the limb and a footrest to support the operative leg. In cases requiring lower flexion angles, a trauma bump may be placed beneath the knee to optimize exposure and positioning. Fluoroscopy setup is critical for accurate imaging. For medial osteotomies, the C-arm is positioned on the operative side, while for lateral osteotomies, it is positioned on the nonoperative side to provide optimal visualization and minimize parallax errors. A tourniquet is typically used at the discretion of the surgeon, and tranexamic acid may be administered for assistance with hemostasis.

A standard approach for medial tibial osteotomy involves an inverted L-shaped incision is made on the medial tibia to access the osteotomy site. The pes anserinus tendons are mobilized and protected during the osteotomy, while the MCL is protected by retracting it posteriorly and ensuring it is not detached during the osteotomy. A neurovascular retractor is placed posterior to the tibia after palpating the fibula head from medial to lateral, which helps protect the popliteal neurovascular bundle and other posterior structures from injury during the osteotomy.

In cases requiring femoral osteotomy, medial or lateral approaches can be utilized based on pre-operative planning for opening or closing wedges for either varus or valgus deformity. Standard approaches to the medial and lateral distal femur are used to perform these osteotomies, and care is taken to protect the neurovascular structures. Because patient-specific guides are customized to the patient’s unique bony anatomy, thorough exposure and subperiosteal dissection must be performed to ensure the guides can be placed accurately on the bony surface without soft tissue interposition. In some cases, abnormalities like osteophytes must be removed to ensure guide fit.

After the guide is positioned appropriately, the guides can be held with temporary fixation such as screws or wires. These often have pre-determined lengths that help prevent violating the far cortex. Fluoroscopy can then be used to confirm correct positioning of the guide and compare its intraoperative position with the pre-operative template. Once proper cutting guide placement is confirmed, osteotomy cuts are performed using different techniques depending on the system being utilized. Some systems use drill bit technology for opening HTO, allowing precise control over the cut depth and reducing the risk of hinge fractures [37]. Other systems utilize an oscillating saw blade through the guide, with a predetermined blade depth calculated during pre-operative planning to ensure accurate resection while preserving the integrity of the far cortex. Following the cut or cuts, fluoroscopy is used to verify correct positioning and confirm a far cortex bony hinge. At this point, the wedge of bone can be removed for closing wedge osteotomies, or the gap can be opened and checked with the help of other printed wedge guides in opening wedge osteotomies. Once this osteotomy is completed, an intra-operative mechanical alignment check can be performed, if desired, using a radiopaque marker such as an electrocautery cord or metallic rod. However, this alignment check is generally less necessary with PSI compared to the freehand technique given the inherent accuracy and customized guides used.

Drill holes for the plate can then be made through a guide, and the appropriate length of each screw, which was calculated preoperatively using the CT scan and software, can be confirmed. For opening wedge osteotomies, bone grafting is typically required when the correction exceeds 10°, as it provides additional stability and promote bone healing. For smaller corrections, the use of bone grafts is at the surgeon’s discretion, though it is commonly implemented to enhance stability. Plates used for closing wedge osteotomies can be lower profile and smaller, as the bone surfaces are brought together, reducing the need for more robust hardware. Finally, the plate is secured, and the wound is closed in a standard fashion, ensuring proper soft tissue management and hemostasis.

In some cases, patients may require concomitant procedures alongside osteotomy, such as osteochondral allograft transplantation, meniscus root repair or transplantation and/or ligament reconstruction, and thus, the osteotomy must be performed with these additional procedures and sequences in mind [38–41]. One major advantage of PSI in this context is its ability to facilitate precise preoperative planning for complex procedures to avoid tunnel convergence, such as when drilling multiple tunnels for a meniscus root repair with and concomitant ACL reconstruction [42].

Some tips for success using PSI include:

- Bring pre-operative 3D models or printed templates to the OR for reference throughout surgery.
- Ensure guides have appropriate labels including side (left or right), degree of correction, and patient identifiers.
- Expose bony landmarks adequately and protect neurovascular structures.
- Verify the location of the guide with both anatomical landmarks and with fluoroscopic imaging to ensure proper placement prior to making cuts.

### Advantages and Limitations of PSI

PSI guides are unique to a patient’s bony anatomy, and pre-operative planning allows surgeons to determine the location and depth of cuts, size of wedge, and screw sizes prior to surgery, which research has demonstrated increases precision, decrease OR time, and fluoroscopy exposure.

### Accuracy of Correction

PSI guides ideally improve the accuracy of planned osteotomies by setting a defined starting point and angle for the bone cuts and defining the planned gap size. Victor et al. published one of the earliest studies on the use of PSI for HTO and DFO and found that in a 14-case series the accuracy of post-operative correction was 0° ± 0.72 mFTA and all cases were within +/- 1° of the planned coronal plane correction, while a study by Munier et al. found < 2° of difference between planned and achieved correction in 19 out of 20 coronal or sagittal corrections [16, 25]. A large case series published by Chaouche et al., including 100 opening-wedge HTO cases, found an accuracy of 1.0° ± 0.9 for mFTA, 0.5° ± 0.6 for mMPTA, and 0.4° ± 0.8 for tibial slope [43]. Another series by Zaffagnini et al. found in 25 patients undergoing HTO with PSI, the mean difference between planned and achieved HKA and PTS was 2.1° ± 2.0° and 0.2° ± 0.4°, respectively [44]. Studies examining more complex procedures, including double-level osteotomies around the knee, have also reported successful and accurate use of PSI [45].

Studies comparing PSI to traditional freehand techniques have also found a superiority in correction accuracy with PSI use. Pérez-Mañanes et al. found that between 20 control patients using traditional techniques for opening wedge HTO compared with 8 cases using PSI, the mean difference between planned and post-operative wedge angle was 1.1° (0°–2.8°) using the conventional technique and 0.5° (0°–1.2°) using PSI [35]. Similarly, Arnal-Burró found in 12 DFOs that the mechanical axis deviation in the coronal plane was, on average, 0.28° mFTA (ranging 0°–1°) with PSI and 1.8° mFTA (ranging 0°–4°) without PSI [36]. Kim et al. found in 20 cases of HTO that the mean absolute difference between planned and achieved correction in the PSI group was 2.3% ± 2.5 to the target point in the weight-bearing line, compared to the conventional technique group of 6.2% ± 5.1 [46]. In a retrospective study of 49 patients undergoing medial opening-wedge high tibial osteotomy with PSI versus 38 with the conventional technique, Fayard et al. found that accuracy of ± 2° in the HKA was achieved in 44 (90%) in the PSCG group and 24 (65%) in the standard group [47]. Recently, a systematic review of 14 studies of PSI for medial opening wedge HTO found the operative accuracy to be within 0.6° of the pre-operative plan on coronal and sagittal planes, as well as an average error within 0.8° of the planned hip-knee correction angle [48].

In addition to precision, Aman et al. reported that rates of correction outliers ranged from 0 to 25% across eight studies utilizing 3D-printed PSI [49]. Although few studies have reported outlier rates on conventional opening wedge HTO, several have reported rates above 30%, with outliers correlating with poor patient-reported outcomes and the deterioration of knee function [50–52]. A 2020 meta-analysis found insufficient evidence to determine if PSI could reduce post-operative outliers compared to traditional techniques, but the rate in the PSI group was reported to be 15% [50].

Despite many articles in the literature supporting greater precision of correction with PSI, several studies have challenged this idea. A study by Abdelhameed et al. found in a retrospective review of patients undergoing knee osteotomies of 50 patients with freehand and 41 patients with PSI technique that there were no statistically significant differences between the target correction and the obtained correction in both groups for any radiologic measurement of alignment [53]. The authors concluded that in experienced hands PSI may not offer any significant benefit. In a study by Tardy et al., 126 patients were prospectively divided into conventional, navigation, and PSI groups. The authors found that in terms of precision, PSI was only significantly improved compared to navigation and not the conventional technique [54].

### Operative Time and Fluoroscopy Exposure

Several studies have included intra-operative variables that may improve with PSI, including operative time and number of fluoroscopy shots taken. Studies have typically found that PSI decreases both OR time and fluoroscopy used intra-operatively. Mao et al. observed an overall reduction in OR time with PSI of 16.8 min during medial opening wedge HTO, while Pérez-Mañanes et al. reported a reduction of 31 min, and Stimolo et al. found a difference of 10.17 min [35, 55, 56]. Similarly, Arnal-Burró et al. found that PSI reduced OR time by 32 min [36].

Regarding fluoroscopy exposure, Mao et al. found an average of 2.8 fewer fluoroscopy exposures using PHI compared to conventional techniques [55]. Stimolo et al. found an overall reduction of 7.41 shots for PSI [56], while Pérez-Mañanes et al. found that the average fluoroscopic image count was 55 for conventional and 8 for PSI, a reduction of 47 [35].

### Cost

A current limitation of PSI is its associated cost. 3D printing is associated with a considerable upfront cost. However, because 3D guides have been observed to decrease operating time, and costs associated with complications may be avoided at a higher rate, the cost 3D printing may be justifiable [57]. Arnal-Burró estimated in their study that cost savings from decreased OR time using PSI was equivalent to €415/procedure, even after considering the added costs of pre-operative CT scan and materials for PSI [36]. Depending on the infrastructure or companies used during preparation, associated pre-operative imaging, design, and processing costs are likely highly variable across institutions.

### Patient Reported Outcomes

While many studies have looked at the provision of correction and intra-operative variables with PSI for knee osteotomies, some have also investigated whether these benefits may translate into superiority in patient-reported outcomes. Research has shown that patient-reported outcomes are improved after knee osteotomies using conventional techniques and PSI [43, 58]. Using PSI, Chaouche et al. observed significant improvements in Knee Injury and Osteoarthritis Outcome Score (KOOS) Pain, KOOS symptoms, KOOS ADL, KOOS, sports/rec, KOOS QOL, and UCLA activity scale scores compared to pre-operative scores [43].

Several studies also report direct comparison between PSI and traditional techniques. In a comparative study between PSI and freehand technique for HTO, Gao et al. found that clinical and functional American Knee Society clinical scores were significantly better in the PSI group at 3 and 6 months postoperatively but that both groups had equivalent scores at final follow-up [59]. Similarly, in a prospective study of 18 patients who underwent medial open wedge high tibial osteotomy using PSI versus 19 who underwent a traditional technique, the authors found significantly higher subjective IKDC scores and Lysholm scores in the 3D-printed group at the 3-month follow-up but not significantly different at other time points [55]. Abdelhameed et al. found in a retrospective review of patients undergoing knee osteotomies of 50 patients with freehand and 41 patients with PSI technique that there were no statistically significant differences in any of the 2-year follow-up KOOS sub-scores [53]. While studies support the clinical efficacy of using PSI for knee osteotomies, there may not be any long-term clinical benefit in patient outcomes, however more research is needed in this area.

### Complications

Overall, complications after knee osteotomies using PSI are low and range between 0 and 13% [9, 16, 25, 43, 47, 55, 59–62]. Complications using PSI are similar to those after knee osteotomies using traditional techniques and include tibial slope changes, hinge fractures, infections, delayed union, and non-union [47]. Thus far, comparative studies also report no significant difference in the complication rate between PSCG and standard techniques [16, 25, 26, 59].

### Drawbacks of PSI

While PSI appear to have several advantages compared to traditional osteotomy techniques, this must be weighed with their limitations. Some of the disadvantages of PSI include increased lead time to surgery to wait for manufacturing of PSI, larger incisions and more soft tissue dissection may be needed to fit guides onto bone, there may be increased pre-operative time spent by the surgeon confirming the guide design, there may be less intra-operative flexibility with cuts, and finally the implant cost is typically more than traditional methods. These limitations must be weighed against advantages discussed previously.

### Learning Curve with PSI

As with any new technique, there is a learning curve for surgeons adopting PSI. However, research thus far has demonstrated that unfamiliar surgeons can learn PSI quickly. In a study of a single surgeon’s experience with 12 consecutive cases of HTO using PSI versus conventional technique, Stimolo et al. found that after 6 cases using PSI, the surgeon progressed from the learning phase to the proficiency phase based on cumulative sum analysis. In comparison, the surgeon remained in the learning phase after 12 cases of HTO using traditional technique [56]. Jacquet et al. observed three surgeons gaining experience with PSI and found that it took an average of 8 cases and 9 cases for a surgeon to decrease their anxiety levels and number of fluoroscopy images, respectively, and no differences in accuracy were noted with the initial cases [63].

### Evolving Applications and Future Directions

The future of PSI will likely include optimizing workflow with new technologies such as artificial intelligence (AI). Studies have demonstrated the ability to incorporate AI into deformity analysis and pre-operative planning with success [64–67]. Recently, Miyama et al. demonstrated that with AI use in pre-operative planning for HTO, there were error rates within 1.5°, inter-rater correlation confidence (ICC) values were comparable to human surgeons with superior intra-rater reliability, and the analysis was performed in less than a second [68]. This technology may assist with operations that require more complex preoperative planning, such as those involving multiligamentous knee injuries (MLKIs) to avoid screw/tunnel convergence. These surgeries are known to be highly individualized procedures requiring precise reconstruction to avoid recurrence and revision [69, 70]. Another promising avenue for future advancement of PSI is increasing its level of modularity. The use of interchangeable segments in existing components can be integrated with PSI to improve intra-operative flexibility, further improving the ease of implant fitting and revision [71, 72]. Modularity also serves to decrease implant inventory and thus operative time [72]. Ultimately, some of the disadvantages of PSI, including the time spent designing and confirming implants for the surgeon and manufacturing team, may be made more efficient over time.

## Conclusion

PSI has emerged as a promising tool for improving the accuracy and efficiency of knee osteotomies. Research has demonstrated increased precision in deformity correction, reduced operative time, and decreased fluoroscopy exposure with similarly improved patient outcomes compared to traditional techniques. While there may be associated costs and a learning curve in adopting PSI, these tools may help surgeons optimize deformity correction, especially in complex cases. Future advancements in PSI, such as improvements of in-house manufacturing cores or the integration of artificial intelligence for pre-operative planning, may further enhance workflow and increase adoption among surgeons.

## Data Availability

No datasets were generated or analysed during the current study.

## Key References

Victor J, Premanathan A. Virtual 3D planning and patient specific surgical guides for osteotomies around the knee: a feasibility and proof-of-concept study. The bone & joint journal. 2013;95(11_Supple_A):153-8.

- This landmark feasibility study was the first to demonstrate that virtual 3D planning and PSI could be safely applied to osteotomies around the knee. It laid the foundation for the modern integration of patient-specific instrumentation in deformity correction.

Munier M, Donnez M, Ollivier M, Flecher X, Chabrand P, Argenson J-N, Parratte S. Can three-dimensional patient-specific cutting guides be used to achieve optimal correction for high tibial osteotomy? Pilot study. Orthopaedics & Traumatology: Surgery & Research. 2017;103 (2):245 − 50.

- This study validated the use of PSI in correcting deformities in both coronal and sagittal planes. It demonstrated excellent accuracy (< 2° deviation from plan) and set a precedent for multiplanar planning in high tibial osteotomies.

Chaouche S, Jacquet C, Fabre-Aubrespy M, Sharma A, Argenson J-N, Parratte S, Ollivier M. Patient-specific cutting guides for open-wedge high tibial osteotomy: safety and accuracy analysis of a hundred patients continuous cohort. International orthopaedics. 2019;43:2757-65.

- This large cohort of 100 patients confirmed that PSI is both safe and accurate in clinical practice, with a high degree of correction accuracy and a low complication rate.

Arnal-Burró J, Pérez-Mañanes R, Gallo-del-Valle E, Igualada-Blazquez C, Cuervas-Mons M, Vaquero-Martín J. Three dimensional-printed patient-specific cutting guides for femoral varization osteotomy: do it yourself. The Knee. 2017;24 (6):1359-68.

- This study extended PSI application to distal femoral osteotomies and demonstrated that 3D-printed guides significantly improved correction accuracy and decreased operative time, reinforcing the versatility and efficiency of PSI.

Dasari SP, Hevesi M, Mameri E, Ferrer-Rivero R, Fortier LM, Jackson GR, et al. Patient-specific instrumentation for medial opening wedge high tibial osteotomies in the management of medial compartment osteoarthritis yields high accuracy and low complication rates: A systematic review. Journal of ISAKOS. 2023;8 (3):163 − 76.

- This systematic review consolidated data from 14 studies and confirmed that PSI improves alignment accuracy while maintaining a low complication profile.

Mao Y, Xiong Y, Li Q, Chen G, Fu W, Tang X, et al. 3D-printed patient‐specific instrumentation technique vs. conventional technique in medial open wedge high tibial osteotomy: a prospective comparative study. BioMed Research International. 2020;2020 (1):1923172.

- This study demonstrated a reduction in operative time and radiation exposure with PSI. It supports the intraoperative efficiency of PSI while maintaining precision and safety.

Tardy N, Steltzlen C, Bouguennec N, Cartier J-L, Mertl P, Batailler C, et al. Is patient-specific instrumentation more precise than conventional techniques and navigation in achieving planned correction in high tibial osteotomy? Orthopaedics & Traumatology: Surgery & Research. 2020;106 (8):S231-S6.

- This study compared PSI, navigation, and conventional techniques and found PSI to be superior to navigation, though not universally better than conventional methods, emphasizing the importance of surgeon experience and context.

## Declarations

### Study Location

This work was performed at Stanford University Medical Center, Stanford, CA.
